# Rab23 has a novel role in the trafficking of Kif17 to the primary cilia

**DOI:** 10.1186/2046-2530-4-S1-P18

**Published:** 2015-07-13

**Authors:** YS Lim, BL Tang

**Affiliations:** 1 Department of Biochemistry, National University of Singapore, Singapore; 2 NUS Graduate School of Integrative Sciences and Engineering, National University of Singapore, Singapore

## 

Rab GTPases are regulators of the intracellular vesicular transport and interorganelle protein trafficking of both endocytic and secretory pathways. The small GTPase Rab23 is an antagonist of the **S**onic **h**edge**h**og (Shh) signaling pathway during mouse embryonic development, but its exact role and mode of mechanism have remained elusive. Since modulation of Shh signaling depends on normal functioning of primary cilia, and Evi5L's overexpression (Rab23's RabGAP) led to decreases in primary ciliogenesis, Rab23 likely has a role at the cilium. We found Rab23 wild-type and constitutively active Rab23Q68L mutant enriched at the primary cilia. In testing the effect of Rab23's manipulations on the ciliary localization of several known ciliary cargoes, ciliary localization of a kinesin-II motor protein Kif17 turned out to be disrupted by overexpression of dominant negative Rab23S23N and in Rab23 depleted cells. In addition, Kif17's ciliary mislocalization could be rescued after expression of a non-degradable wild-type Rab23 and FRAP experiments showed that Rab23 silenced cells exhibited reduced recovery of ciliary Kif17 after photobleaching. Co-immunoprecipitation studies further revealed that Rab23 exists in a tripartite complex with Kif17 and Importin β2 (Kif17's ciliary import carrier), implying that Kif17 requires binding to regulatory proteins like Rab23 for its ciliary transport. Although a ciliary-cytoplasmic gradient of nuclear Ran is necessary in regulating Kif17's ciliary import, both small GTPases Rab23 and Ran appear to have independent roles in the ciliary entry of Kif17. Our findings have uncovered a hitherto unknown effector of Rab23 and demonstrated how Rab23 could mediate Kif17's trafficking to the primary cilium.

